# Supplementary material for: Urgent need for consistent standards in functional enrichment analysis
Source: PLoS Comput Biol. 2022 Mar 9;18(3):e1009935. doi: 10.1371/journal.pcbi.1009935 (PMC8936487; doi:10.1371/journal.pcbi.1009935)
Supplement: Attachment — Submitted filename: Reviewer comments v2.pdf [file pcbi.1009935.s004.pdf]

Dear Editor,

Thank you for the opportunity to resubmit our article.

The largest changes made were to remove any mention of the combined/separate ORA issue raised by reviewer 2. A panel was removed from Figure 2 (previously labeled 2K). Moreover, Figure 4 has been substantially changed. Fig 4A is designed to demonstrate the effect of ignoring p-value adjustment. ORA combined/separate has been removed from panels 4D and 4E. This makes for a more focussed article highlighting the three major issues outlined in the abstract.

We have provided two versions of the manuscript, one clean and the second with changes highlighted. We have attended to the comments and suggestions of both reviewers as detailed below.

Regards,

Mark Ziemann PhD

Reviewer #1

Enrichment analysis is widely used to interpret high-throughput omics data in terms of functional categories and pathways. In this study the authors laboriously surveyed 1630 genomics papers to assess whether enrichment analyses are conducted properly. Of these papers, 186 were studied in detail as they were cross checked by at least two team members. Their main finding is that only 15% of screened analyses are conducted and documented properly. Main issues in the rest are not using or reporting background genes (95%), failure to correct for multiple testing (43%), pooling up and down regulated genes. The author also analyzed an RNA-seq data set to demonstrate that how enrichment results differ when analyses is done inappropriately. As the first large-scale survey of its kind, this article sounds the alarm, once again, on the widespread abuse of statistics by the biomedical and genomics research community. The incompetency and the carelessness call for urgent action to improve reproducibility.

Thank you for the positive feedback and useful comments.

1. Fig.3b and 3f probably could be more effectively using boxplots/violin plots by treating analysis score as categories. Overlapping dots, even with color gradient, are hard to interpret.

R1P1 response: We agree that the overlapping points are hard to interpret, so we have swapped these panels for box plots as suggested.

2. Annotation databases for some online tools are not updated frequently. For example, the database for DAVID is last update in 2016. See https://david.ncifcrf.gov/content.jsp?file=release.html The author could discuss how obsolete annotation affect the results of enrichment analysis.

R1P2 response: We think the use of up-to-date tools and databases is an important consideration. This point was also raised by reviewer 2. We have added the following passage to the introduction (bottom of page 3) which highlight this point and touches on the history behind the GO and KEGG consortia:

“The validity of functional enrichment analysis is dependent upon rigorous statistical methods as well as accurate and up-to-date gene functional annotations. Two of the most frequently used databases of gene annotations are Gene Ontology (GO) and Kyoto Encyclopedia of Genes and Genomes (KEGG). Both databases emerged around the time of public release of the the first eukaryotic genomes, with the aim of systematically cataloging gene and protein function [1,2],”

(We also note that DAVID just released an update in December 2021.)

Reviewer #2

Wijesooriya et al. Urgent need for consistent standards in functional enrichment analysis

The Ziemann team address a fundamental scientific issue in the field of bioinformatics. Building on earlier commentaries and case examples they systematically address and quantify the extent of the 'erroneous pathway p-value issue' in biomedical research. Their conclusions are not surprising to this reviewer, yet these results make urgent and essential reading for all reviewers, journal editors and biomedical researchers working with OMIC data.

Thank you for the many useful comments and feedback.

1. There is one main and easy to solve flaw in their article and that pertains to an argument about combining or otherwise up and down-regulated genes. There is no logic or rule for this and in biological pathways combining is completely acceptable, as you can have both positive and negative regulators in a list from one pathway, differentially expressed in the opposite direction. Thus the interpretation of the statistical work by Hong G et al 2014 is wrong – unsurprisingly as they understand little biology based on a reading of their article.

   Thus an up-regulation of a positive actor or down-regulated of a negative actor – in the same pathway - can equate to the same biochemical outcome. Splitting lists also actually leads to two other problem. First is that with some technologies detecting down-regulation is more difficult (signal related) and second is that related to gene list size. Its well appreciated that small lists, especially if enrichment ratios + pvalues, and/or boot strapping are considered, are not sensitively profiled. Splitting a biologically relevant list into up and down impacts on this issue in an unpredictable manner, depending on gene list size and content. This particular section needs re-written or deleted as its wrong. Your results – see below – actually stumble on this issue.

   R2P1 response: Thank you for this thoughtful comment. I see that combined and separate are simply two different null hypotheses and are equally valid. For this article we will focus on the issue of background, FDR and missing details, so we will delete those parts of the results and discussion. Figure 4 is now totally focussed on the effect of ignoring p-value correction and improper background, and makes for a more focused set of findings consistent with the take home message in the abstract.

Specific comments. The majority of my comments are minor and relate to ensuring clarity of message and identification of any statement that could be misconstrued (or is not entirely accurate).

2. I would briefly mention the history behind the Gene Ontology Consortium and their project to provide context as to how this catalogue of processes/pathways emerged. http://geneontology.org/docs/introduction-to-go-resource/

   It's possibly informative to reflect on the fact that this grew out of a need to catalogue the genome (with the incorrect assumption that all genes were equally characterizable in a genome wide study). So perhaps they never thought about a variable detectable background.

   R2P2 response: To address this we have added the following passage to the end of the first paragraph of the introduction (bottom of page 3):

“.. Therefore, the validity of functional enrichment analysis is dependent upon rigorous statistical methods as well as accurate and up-to-date gene functional annotations.”

And a new second paragraph (top of page 4):

“Two of the most frequently used databases of gene annotations are Gene Ontology (GO) and Kyoto Encyclopedia of Genes and Genomes (KEGG). Both databases emerged around the time of public release of the the first eukaryotic genomes, with the aim of systematically cataloging gene and protein function [1,2],”

3. You should probably mention that the Gene Ontology Consortium current link to their “10 tips for GO” does not mention the word ‘background’ even once.
   https://journals.plos.org/ploscompbiol/article?id=10.1371/journal.pcbi.1003343

   This is a paragraph from Chapter from the ‘The Gene Ontology HandBook’. 2017. They STILL don’t correctly explain the background bias issue.

   “Such large changes in GO annotations can affect GO enrich- ment analyses, which are sensitive to the choice of background distribution (Chap. 13 [3]; [20]). For instance, Clarke et al. [21] have shown that changes in annotations contribute significantly to changes in overrepresented terms in GO analysis. To mitigate this problem, researchers should analyze their datasets using the most up-to-date version of the ontology and annotations, and ensure that the conclusions they draw hold across multiple recent releases. At the time of the writing of this chapter, DAVID, a popular GO analysis tool, had not been updated since 2009”

   R2P3 response: The handbook does describe the background issue (Chapter 13 p175-177), however it is not communicated in a clear and direct way which is easily understood by the novice user. We have alluded to this in the Discussion (page 13) as an explanation as to why such problems are so common.

   “These shortcomings are understandable, as some popular tools do not accommodate background gene lists by design (eg: [25]). Moreover some user guides gloss over the problems of background list and correction for multiple testing (eg: [26]), while other guides are written in such a way that they are difficult for the novice statistician to comprehend (eg: [27]). Certainly the inconsistent nomenclature used in different articles and guides makes it difficult for beginners to grasp these concepts. Unfortunately, some of the best guides for enrichment analysis are paywalled (eg: [5,7]) which limits their accessibility. With this in mind, there is a need for a set of minimum standards for enrichment analysis that is open access and written for the target audience (life scientist with little expertise in statistics)”

4. P3 ‘developed to summarise gene profiles into simplified functional categories’

   Try – ‘developed to summarise regulated gene expression profiles into simplified functional categories’

   R2P4 response: Fixed

   P4 ‘A statistical test is performed to ascertain whether the number of DEGs belonging to a particular gene set is higher than that expected by random chance, as determined by comparison to a background gene list. These ORA tools can be stand-alone software packages or web services, and they use one or more statistical tests (eg: Fisher’s exact test, hypergeometric test) [1,2]’.

I have two comments here.

5. Most ignore the enrichment ratio (which helps inform about bias related to very large (usually uninformative) gene sets) e.g. significant p-value and ER of 1.1 is not a meaningful result (given all the sources of bias). I would explicitly mention that ‘is ideally both a significant adjusted p-value and a robust enrichment ratio (ratio of regulated genes to total genes in that group) is sought’.

   R2P5 response: This is indeed true. We have alluded to this in the introduction in a new passage on page 5:

   “Another issue is the reporting of statistically significant enrichments, where the observed effect size (enrichment score) is so small it is unlikely to have any meaningful biological effect [17].”

6. Second point is that I believe it is essential to mention the published objections to use of Fisher’s exact test (or worse still hypergeometric test). In that they assume independence and for members of gene sets that not true. I would state there are “general concerns” about the weakness of the primary statistical methods. The reason I mention this is some colleagues won’t engage on the larger issue of background bias or lack of FDR use, because they object to the primary statistical method. Better to state you recognise their concerns with a sentence and citation.

   A good review article to cite on the GO stats would be https://www.nature.com/articles/nrg2363

   Also note this sentence from the review.

   “In practice, a term would need to have a raw p-value less than 4 x 10−7 for it to be significant at the 1% significance level. Other corrections, such as Holm's26 and false discovery rate27, are less conservative but loss of power cannot be completely avoided (see Refs 28,29 for further reviews). Hence, as a general rule, one can increase the power of the statistical analysis by performing the fewest possible number of tests.”

   This review deals with things are a theoretical level and do not do the essential work you provide in your article – namely the scale for the problem. It is worrying however how little of this review in 2008 has been recognised, compared with the use of GO tools.

   R2P6 response: Thank you for raising this. We have included citations to Rhee et al [5] where appropriate. In the introduction (page 5) we have mentioned very briefly these limitations of enrichment analysis:

   “Although these are powerful tools to summarize complex genomics data, there are limitations. For example many ORA and FCS approaches assume independence between genes, which is problematic, as genes of the same functional category are somewhat more likely to have correlated gene expression [14]. There is an ongoing debate as to whether ignoring non-independence is a reasonable simplifying assumption in functional enrichment analysis [15,16].”

7. Hammering home that a p-value of 1x10-6 BEFORE correction is likely to required will provide some reality check to those using GO tools and the designers of software – as they are the MAIN problem today. If your tool does not give good results people will not use them.....thats clearly an issue.

   Secondly, many select EVERY GO class, or GO + KEGG + XYZ when using online tools - This is clearly a flawed approach, yet software tools enable this mistake.

R2P7 response: These are important considerations, although in our article here we are trying to focus on the most egregious problems which are the easiest to remedy. We think the software design considerations and GO + KEGG + XYZ problem would be best described in a separate, dedicated “best practices” article.

The main point to make here is that users can avoid the worst errors with FDR adjustment and using the correct background. The nominal p-value threshold suggestion has a lot of merit but will not be consistent across algorithms and as such we cannot make sweeping recommendations as the tool ecosystem is so diverse.

8. A further point that you may wish to consider specifically in relation to microarrays and RNAseq. The modern microarray provides greater coverage (See Sood et al Nucleic Acid research 2016 and Timmons et al Aging Cell 2019 and supplement), and are more sensitive (See Fig 2e, Peters TJ Bioinformatics 2019) when profiling individual human tissues than RNAseq.

   RNAseq has a library (PCR bias and incomplete nature means a gene can not ‘appear’) and also a serious reproducibility problem (See Supplement S22 of the Sequencing Quality Control Consortium, Nature Biotechnology 2014 – 32(9)) meaning that the certainty of the background becomes less clear than for a modern array processed with modern methods. See Mandelboum et al Plos Biology 2019).

   Single Cell RNAseq becomes an even greater issue – here coverage is normally between 2000-5000 genes and heavily biased for high abundance genes esp. mitochondrial (See the Gene Ontology data buried in the supplement of Mereu et al Nature Biotechnology June 2020).

   In short, for sequencing experiments the GO/Pathway background issue just became a far greater problem. This is important.

   R2P8 response: Thank you for this important point. Indeed the lack of RNA-seq coverage of lowly expressed genes is a major contributor to results in Fig 4, which is less of a problem for microarray studies. We have mentioned this in the discussion with reference to Sood et al [24] (page 12):

   “In the seven RNA-seq examples examined here, using the inappropriate whole genome background gave results that were on average only 44% similar to results obtained using the correct background (Fig 4E).

   “The severe impact of selecting the wrong background on RNA-seq functional enrichment results is due to the fact that in RNA-seq, only a small fraction of all annotated genes are detected. Table 2 indicates that only ~38% of genes are detected in the seven examples. In contrast, a modern microarray detects a larger proportion of genes [24], so the effect of using a whole genome background is less severe.”

   In the introduction, we also mention the impacts on single-cell RNA-seq and proteomics:

   “However the problem becomes more acute when the proportion of measured genes/proteins is small, for example in proteomics and single-cell RNA-sequencing where only a few thousand analytes are detected.”

9. Page 6 “From these, we initially selected 200 articles for detailed methodological analysis.”

   Please clarify why and how you selected the 200 for the summary chart arm of your flow chart.

R2P9 response: These articles were selected totally at random using the Unix command “shuf”. The corresponding results and methods sections have been amended accordingly:

Results (bottom of page 7): “From these, we randomly selected 200 articles for detailed methodological analysis.”

Methods (page 15): “We initially sampled 200 of these articles randomly using the Unix “shuf” command. We then collected the following information from the article …”

10. I am curious that so few Nature Communication articles appear in your analysis. This is the largest single source of OMIC papers I encounter and almost without exception there is no clear methods and obvious flaws in the GO/Pathway analysis. The other journals that have a high frequency of flawed GO/Pathway analysis are the American Journal of Physiology family of journals.

    In that sense Fig S1A is not helpful as its not an unbiased or full representation of the sources of the problem – and might give the wrong impression.

R2P10 response: We encountered one Nat Comms article in the initial screen of 200 articles and five in the secondary screen. It may well be the largest single source of articles that the reviewer comes across because it is an attractive journal to submit to (high JIF). However with an acceptance rate of only 7% most articles get rejected. In terms of published articles describing enrichment analysis, those multidisciplinary journals like PLoS One, Sci Rep and PeerJ are the most prolific. These journals also have much lower rejection rates (approximately 50-60%).

Consistent with your experience and despite peer review, these published Nat Comms articles exhibited many problems as you can see in the table below:

Pubmed.Central.ID	Journal	Omics.type	Organism	Gene.set.library	GS.version	Statistical.test.used	FDR.Correction	App.used	App.Version	Code.availability	Background.gene.set	Assumptions.violated	Gene.lists.provided
PMC6353904	Nat Commun	Gene expression array	Homo sapiens	Ingenuity Knowledge Base	No	Not stated	No	Ingenuity Pathway Analysis	No	NA	No	Background, FDR	Yes
PMC6445072	Nat Commun	Genotyping array	Homo sapiens	MSigDB, GWAS-catalog	No	Hypergeometric	Yes	FUMA	No	NA	Not stated	Background	Yes
PMC6527683	Nat Commun	Genotyping array	Homo sapiens	Not stated	No	Not stated	Yes	PANTHER	No	NA	Not stated	Background	No
PMC6611870	Nat Commun	Gene expression array	Homo sapiens	KEGG	No	Not stated	No	Not stated	No	NA	Not stated	Background, FDR	No
PMC6616627	Nat Commun	Genome sequencing	Homo sapiens	GO, DisGeNET	No	Not stated	Yes	ToppGene	No	Yes	Not stated	Background	No
PMC6646387	Nat Commun	Proteomics	Homo sapiens	Ingenuity Knowledge Base	Yes	Not stated	No	Ingenuity Pathway Analysis	Yes	NA	Not stated	Background, FDR	No

We agree in part that the proportion of flawed studies shown here is not a true representation of enrichment analysis more broadly, in particular our findings may not be consistent with articles in high ranking journals. In the discussion (page 14) we mention the limitations:

“Many open-access articles examined here are from lower-ranked journals that might not be representative of articles in paywalled journals. The articles included in this study contained keywords related to functional enrichment in the abstract, and it is plausible that articles in higher ranked journals contain such details in the abstract at lower rates. Those highly ranked specialist genomics journals are likely to have lower rates of problematic articles due to more knowledgeable editors and peer reviewers.”

11. P8 During this survey, we noticed some studies grouped up- and down-regulated gene lists together prior to ORA, a practice we were not expecting.

    As mentioned above you have made a mistake in logic, regarding up and down-regulated gene lists and their combination. See above.

    R2P11 response: as above, we have omitted the separate/combined issue from this article.

P11. Figure 3D is remarkable – BMC Bioinformatics being one of the lowest scoring journals. I am glad I resigned from their editorial board 8yr ago (for poor quality editorial processes).

12. P12. The RNA-seq data you analyse using only an FDR filter and with >20% DE raises issues about normalisation if such a large % of genes are genuinely DE. I personally would check implementing a modest FC filter on top of the p-value to enrich in true positives and confirm that you still have only moderate concordance. In short, if you put a lot of 'junk' in, you can not expect reliable data out and its best to avoid any critic of this analysis.

    R2P12: Filtering based on fold change and p-value is sometimes conducted, but there is no consensus on the thresholds to use, which is problematic for novice analysts. For example, what precisely is a "modest FC filter"? In addition (Zhang & Cao; PMID19995439) have argued that filtering by fold change is largely unnecessary.

    Although we didn't quantify the proportion of ORA studies that performed double filtering, my recollection of these papers suggests it was conducted in less than half of the analyses. So in keeping with the majority of the studies evaluated here, we won't include a FC filter.

P12 "Interestingly, 26 gene sets were simultaneously up and downregulated with this approach."

13. This is probably a reflection of the issue of splitting up and down regulated lists as I mentioned. The direction does not come from GO, it comes from your assumption that up means "process up" and vice versa. This is not reliably concluded without detailed inspection as, as stated, loss of a negative regulator results in up-regulation of pathway function. You need to rethink this part of your paper otherwise you are jeopardising the validity of your article. Likewise you need to re think how you cite the flawed conclusions made by Hong 2014.

    14. Hong G, Zhang W, Li H, Shen X, Guo Z. Separate enrichment analysis of pathways for up- and downregulated genes. J R Soc Interface. 2014;11: 20130950.

    R2P13 response: as above, we have omitted the separate/combined issue from this article. We have removed the reference to Hong 2014.

14. Page 15

    "This is despite ORA tools being reported to lack sensitivity when compared to FCS according to previous benchmarking studies [7-9]"

    "Although analyses involving GSEA scored better overall, they were not free of issues. "

    You should probably consider that FCS/GSEA represent a different set of biases (depending on the origin and date of the gene-sets) than ORA GO type analysis. Arguing that FCS is more sensitive is partly a reflection of using the KS statistic and there are strong advocates against the robustness of KS (and several iterations trying to address them). On the other hand being able to show that the accumulation of a large number of small changes in a pathway might be biologically

sound is attractive. I would present + and – rather than just that FCS is ‘better’ based on sensitivity.

R2P14 response: There is a clarification that needs to be made. The second quote is about the methodology scores of the articles that used GSEA, which were on average higher than studies using ORA. This just means that the methodology was better described in those articles and that there were fewer statistical problems. This is not saying that GSEA is better.

The first quote is patently true. When there are only a few DE genes under the FDR threshold, then it is highly unlikely that any enrichment would be significant (lack of sensitivity). This is shown in Kaspi & Ziemann Fig 7A (PMID:32600408), that FCS methods are more robust to noisy data. Zyla et al (PMID:31165139) go so far as to recommend against using ORA in most cases.

I note that Tarca (PMID:24260172) shows that ORA has the potential to be very sensitive, but the procedure they used for classifying DE genes is convoluted and contrived.

So to clarify this point in the manuscript we have amended the Discussion (page 13):

“Although articles that used GSEA obtained higher methodology scores overall, they were not free of issues.”

Methods

15. List that the journal scoring metric is logical but ad hoc, and it is not scaled by the magnitude of impact of each error on the results.

    Human nature is to say “I followed almost all the criteria so I am doing well” when they could miss out the most damaging rule e.g. No FDR and inappropriate background”. I’d state you are not implying that all scoring criteria are equal.

    R2P15 response: We have added a passage to the discussion (page 14) to address this:

    “We also recognise the simplistic nature of the analysis scoring criteria. Clearly, the impact of each criterion is not the same. For example the effect of ignoring FDR is likely more severe than omitting the version number of the tool used. This simplified scheme was used for practical reasons.”

    (As a side note, new Fig 4 shows that inappropriate background is a more severe error than lack of FDR correction [for RNA-seq].)

16. From peer reviewing the number of times I have informed an author that they get “relevant pathways” (to their tissue/biology) because they compare with a genome wide background and that creates fake enriched p-values – only to have this ignored and the fake enriched p-values published, is substantial.

    Defining the correct RNAseq background is particularly challenging as many samples can have essentially zero counts for a particular gene (unrelated to group membership), and yet its called detected by some % call. Proportion calls with groups (block design) is required. You might wish to write something more about the sources background biases or cite from Timmons 2015, that lists them.

    R2P16 response: I have had a similar experience regarding reviews. Preventing “pollution” of the literature with poor studies is the primary motivation for this work. Regarding the RNA-seq

background problem, we have included the following passage in the Discussion (page 13) to lead the reader toward relevant literature.

“There are various approaches to define the background of an RNA-seq dataset [25]. The effect of different filtering methods on differential expression results has been investigated [26], and provides us with some practical recommendations to avoid sampling biases described by Timmons et al [18].”
